# Supplementary material for: Upregulation of Cell Surface Glycoproteins in Correlation with KSHV LANA in the Kaposi Sarcoma Tumor Microenvironment
Source: Cancers (Basel). 2023 Apr 6;15(7):2171. doi: 10.3390/cancers15072171 (PMC10093722; doi:10.3390/cancers15072171)
Supplement: Supplementary file 1 [file cancers-15-02171-s001.zip › cancers-2213874-supplementary.pdf]

Supplementary Materials

Upregulation of Cell Surface Glycoproteins in Correlation with KSHV LANA in the Kaposi Sarcoma Tumor Microenvironment

Sara R. Privatt, Owen Ngalamika, Jianshui Zhang, Qinsheng Li, Charles Wood and John T. West

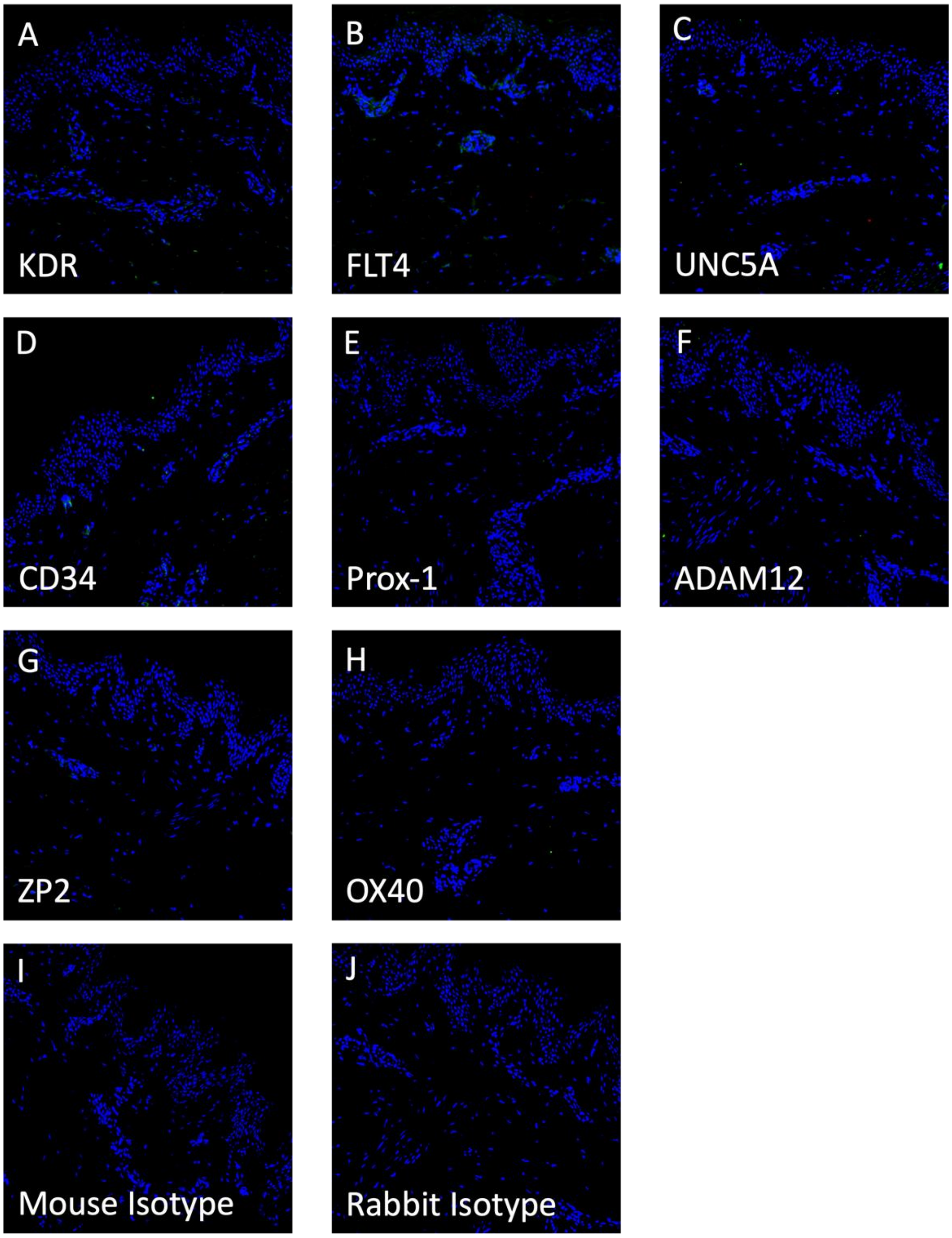

Figure S1. Dual immunofluorescence for all markers in normal human skin tissue. LANA is denoted as red and the surface marker in question is green. Staining of (**A**) KDR, (**B**) FLT4, (**C**) UNC5A, (**D**) CD34, (**E**) Prox-1, (**F**) ADAM12, (**G**) ZP2, (**H**) OX40, (**I**) Mouse Isotype, (**J**) Rabbit Isotype. No LANA staining and minimal staining of the surface markers were observed. Images were acquired at 20X magnification.

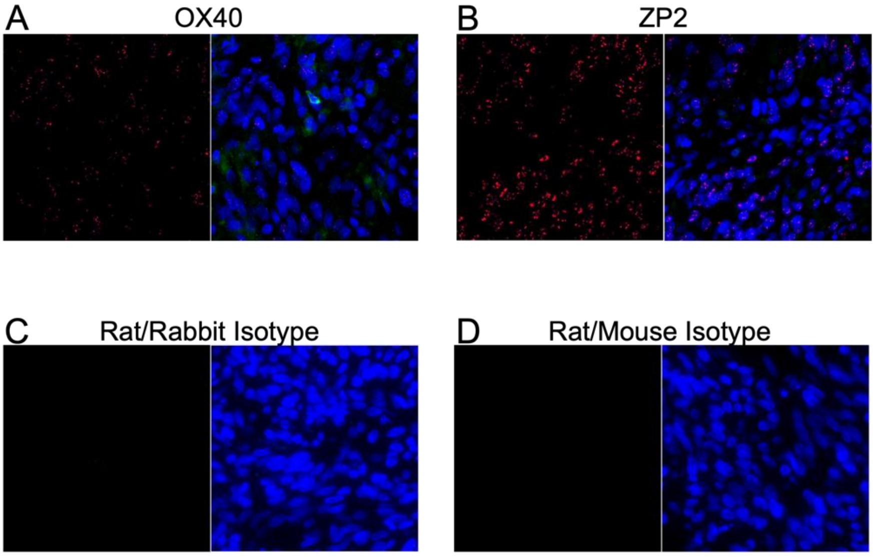

Figure S2. Dual immunofluorescence for OX40, ZP2, and isotype controls of KS tissue. (**A**) OX40; (**B**) ZP2; (**C**) rat and rabbit isotype control; (**D**) rat and mouse isotype control. LANA is denoted as red and the surface marker in question is green. Images were acquired at 20X magnification.

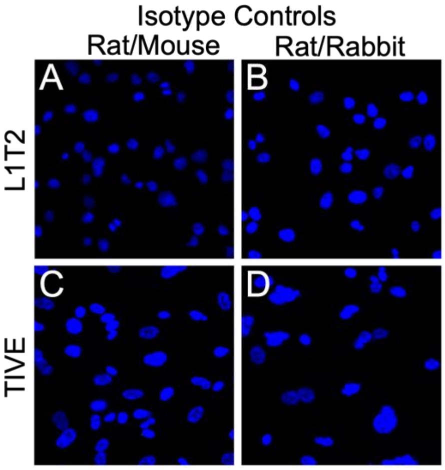

Figure S3. Negative staining of isotype controls in L1T2 and TIVE cells. (**A**) and (**C**) Rat and mouse isotype control; (**B**) and (**D**) rat and rabbit isotype control. Images were acquired at 20X magnification.

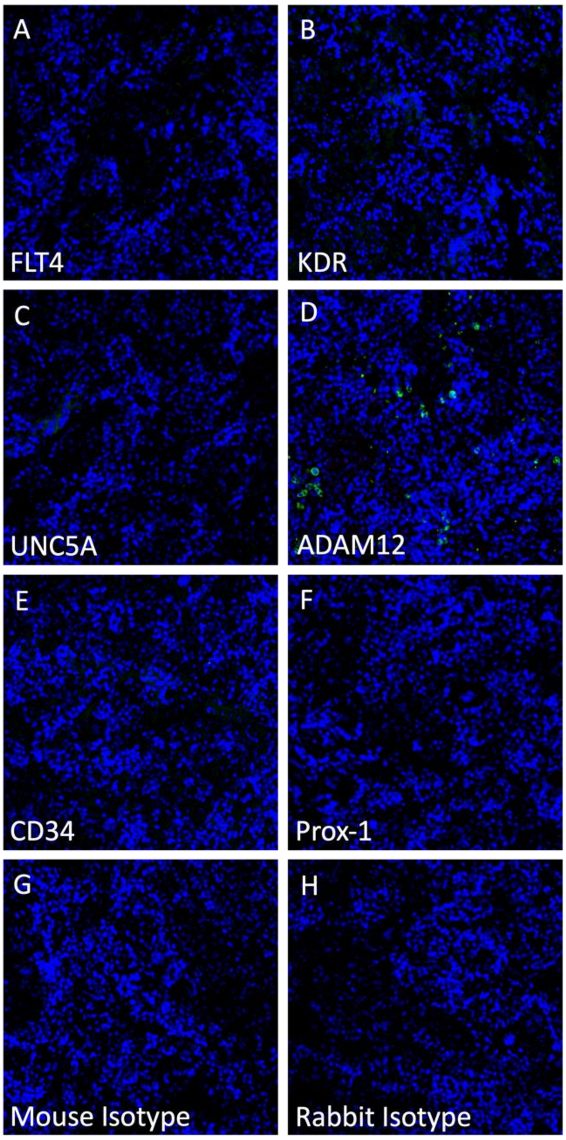

Figure S4. Dual immunofluorescence for all markers in L1T2 derived tumor-bearing mouse spleens. LANA is denoted as red and the surface marker in question is green. Staining of (**A**) FLT4, (**B**) KDR, (**C**) UNC5A, (**D**) ADAM12, (**E**) CD34, (**F**) Prox-1, (**G**) Mouse Isotype, (**H**) Rabbit Isotype. No LANA staining and minimal staining of the surface markers was observed. Images were acquired at 20X magnification.

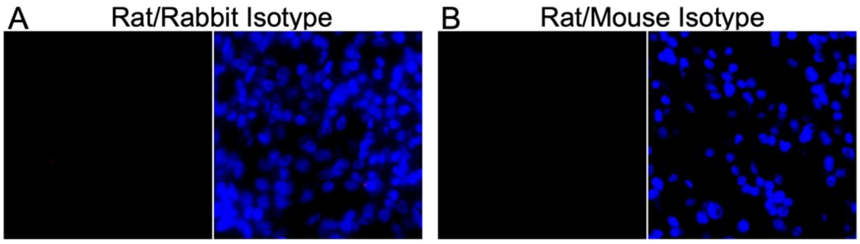

Figure S5. L1T2-derived mouse xenograft isotype controls. (**A**) rat and rabbit isotype control; (**B**) rat and mouse isotype control. Images were acquired at 20X magnification.
